# Novel Virulence Role of Pneumococcal NanA in Host Inflammation and Cell Death Through the Activation of Inflammasome and the Caspase Pathway

**DOI:** 10.3389/fcimb.2021.613195

**Published:** 2021-03-11

**Authors:** Yu-Wen Tseng, Chun-Chi Chang, Yung-Chi Chang

**Affiliations:** Graduate Institute of Microbiology, College of Medicine, National Taiwan University, Taipei, Taiwan

**Keywords:** *Streptococcus pneumoniae*, sialidase, NanA, inflammasome, caspase

## Abstract

*Streptococcus pneumoniae* is one of most deadly Gram-positive bacterium that causes significant mortality and morbidity worldwide. Intense inflammation and cytotoxicity is a hallmark of invasive pneumococcal disease. Pneumococcal NanA has been shown to exaggerate the production of inflammatory cytokines via unmasking of inhibitory Siglec-5 from its sialyl *cis*-ligands. To further investigate the mechanistic role of NanA and Siglec-5 in pneumococccal diseases, we systemically analyzed genes and signaling pathways differentially regulated in macrophages infected with wild type and NanA-deficient pneumococcus. We found that NanA-mediated desialylation impairs the Siglec-5-TLR-2 interaction and reduces the recruitment of phosphatase SHP-1 to Siglec-5. This dysregulated crosstalk between TLR-2 and inhibitory Siglec-5 exaggerated multiple inflammatory and death signaling pathways and consequently caused excessive inflammation and cytotoxicity in the infected macrophage. Collectively, our results reveal a novel virulence role of NanA in pneumococcal pathogenesis and suggest that targeting NanA activity may ameliorate the pneumococcus-mediated inflammation and cytotoxicity in severe invasive pneumococcal diseases.

## Introduction

*Streptococcus pneumoniae* (SPN, pneumococcus) is a common colonizer of the human upper respiratory tract (URT) with an carriage rate of 20-50% and 8-30% in healthy children and adults, respectively (Melegaro et al., 2004; Regev-Yochay et al., 2004; Mccullers, 2006). Invasive pneumococcal diseases such as pneumonia, bacteremia, and meningitis occur when the bacteria spread from the nasopharynx to the lungs, blood, and brain. Over 14.5 million invasive pneumococcal diseases are recorded annually, with a case fatality rate of 11% for children under the age of 5 years and 10-25% for the elderly (Black et al., 2010; Spijkerman et al., 2011; Musher and Thorner, 2014). This bacterium is responsible for over a million deaths annually (Kadioglu et al., 2008; O’brien et al., 2009).

The host recognizes pneumococci and orchestrates immune responses via multiple pattern recognition receptors (PRRs), including the membrane-bound Toll-like receptors (TLRs) and the cytosolic nucleotide-binding oligomerization domain-like receptors (NLRs). Upon activation, these PRRs induce the expression of proinflammatory cytokines at the levels of transcription and post-translational proteolytic processing (Koedel et al., 2003; Malley et al., 2003; Knapp et al., 2004; Opitz et al., 2004; Mcneela et al., 2010; Davis et al., 2011; Witzenrath et al., 2011). Intense inflammation and cytotoxicity is hallmarks of pneumococcal diseases, which contribute to the clearance of bacteria, however excessive activation of the same immune responses is often detrimental to the host (Dockrell et al., 2003; Musher et al., 2004; Corrales-Medina and Musher, 2011).

Siglecs are sialic acid-binding immunoglobin (Ig)-like lectins which broadly express throughout the immune system. Most Siglecs have a cytoplasmic immunoreceptor tyrosine-based inhibitory motif (ITIM) and are considered to play an inhibitory role in the immune system via recruiting the SH2 domain containing protein tyrosine phosphatase (SHP)-1 and SHP-2 (Crocker et al., 2007; Pillai et al., 2012). In static state, Siglecs are ‘masked’ by the *cis*-sialyl ligands expressing on the same cell to initiate an ITIM-mediated suppressive signal, which limits immune cell activation and maintains immunological homeostasis. Removal of sialic acids from the cell surface has been shown to enhance the inflammatory response of monocytes to LPS stimulation (Stamatos et al., 2010). Sialic acid mimetic treatment, which reduces sialic acid expression and subsequently abrogates the interaction between Siglecs and its *cis*-sialyl ligands, drastically lowered the activation threshold of dendritic cells upon TLR engagement (Bull et al., 2017). In contrast, administration of sialidase inhibitor protected mice from polymicrobial sepsis and LPS-induced endotoxemia (Chen et al., 2011; Chen et al., 2014). These observations suggest that the content of surface sialic acids plays a crucial role in controlling immune cell activation.

A broad and direct interaction between Siglec and TLR was identified where Siglec negatively regulates TLR activation (Chen et al., 2014). Mammalian neuraminidase-1 (Neu-1), which translocated to the cell surface upon LPS stimulation, has been shown to disrupt the interaction between Siglecs and TLR-4 and restore the TLR-4 function (Amith et al., 2010; Abdulkhalek et al., 2011; Chen et al., 2014). In addition, we found that bacterial sialidase, NanA, causes exacerbated host inflammation through releasing Siglec-mediated immunosuppression (Chang et al., 2012), although the mechanism by which NanA exerts this immunomodulatory effect is not fully understood. In this study, we demonstrated that NanA-mediated desialylation impairs the Siglec-5-TLR-2 interaction and reduces the recruitment of phosphatase SHP-1 to Siglec-5. This dysregulated crosstalk between TLR-2 and inhibitory Siglec-5 provokes the activation of PRR-related signaling molecules, inflammasomes, and caspases, which consequently results in the excessive inflammation and cytotoxicity of infected host cells.

## Materials and Methods

### Antibodies and Reagents

Antibodies used in this study were listed in **Table 1**. Inhibitors Ac-YVAD-cmk and MCC950 were from Sigma and Z-IETD-FMK was from Enzo Life Sciences. Biotin-conjugated *Erythrina cristagalli* lectin (ECA) and peanut agglutinin (PNA) were from Vector Laboratories. The Annexin V/7-AAD apoptosis kit was from BioLegend.

**Table 1:** Antibodies and primers used in this study.

Antibody	Source	Application
β-actin	Sigma #A5441	WB
ASC	Santa Cruz Biotechnology #sc-271054	WB
Caspase-1	Cell Signaling Technology #3866	WB
Caspase-8	Cell Signaling Technology #9496	WB
Flotillin-1	BD Biosciences #610821	WB
Gasdermin D	Cell Signaling Technology #93709	WB
IKKβ	Cell Signaling Technology #8943	WB
IL-1β	Cell Signaling Technology #12242	WB
LC3A/B	Epitomics #2057-1	WB
p38α MAPK	Cell Signaling Technology #9217	WB
p44/p42 MAPK (Erk1/2)	Cell Signaling Technology #9107	WB
Siglec-5	BioLegend #352002	IP
Siglec-5/14	R&D Systems #AF1072	WB
SHP-1	Santa Cruz Biotechnology #sc-7289	WB
SHP-2	Santa Cruz Biotechnology #sc-280	WB
SOCS3	Origene #TA503055	WB
TLR-2	BioLegend #309702	IP
TLR-2	Cell Signaling Technology #12276S	WB
phospho-AMPKα (T172)	Cell Signaling Technology #4188	WB
phospho-Akt (S473)	Cell Signaling Technology #9271	WB
phospho-(c)Jun (S63)	Cell Signaling Technology #2361	WB
phospho-IKKα β (S176/S180)	Cell Signaling Technology #2697	WB
phospho-JNK (T183/Y185;T221/Y223)	Merck #07-175	WB
phospho-Lck (Y505)	Cell Signaling Technology #2751	WB
phospho-Lyn (Y507)	Cell Signaling Technology #2731	WB
phospho-MEK1 (S218/222)/ MEK2 (S222/226)	Merck #05-747	WB
phospho-MKK3 (S189)/MKK6 (S207)	Cell Signaling Technology #9236	WB
phospho-MKK7/SKK4 (T275)	Merck #36-013	WB
phospho-NF-κB p65(S529)	Epitomics #2884-1	WB
phospho-p38α MAPK (T180/Y182)	Cell Signaling Technology #4511	WB
phospho-p44/42 MAPK (Erk1/2)(T202/Y204)	Cell Signaling Technology #4370	WB
phospho-p70 S6 Kinase (T389)	Merck #MABS82	WB
phospho-PDK1 (S241)	Cell Signaling Technology #3438	WB
phospho-PKCγ (T655)	Merck #07-879	WB
phospho-PKR (T446)	Merck #07-532	WB
phospho-PLCγ1 (Y783)	Cell Signaling Technology #2128	WB
phospho-Shc (Y317)	Cell Signaling Technology #2431	WB
phospho-SHP2 (Y542)	Epitomics #2184-1	WB
phospho-Syk (Y323)	Merck #07-915	WB
phospho-Syk (Y525/526)	Cell Signaling Technology #2710	WB
phospho-Src (Y416)	Merck #05-677	WB
phospho-Src (Y527)	Cell Signaling Technology #2015	WB
IRDye® 800CW Donkey anti-Rabbit IgG (H + L)	LI-COR, Cat#926-32213	WB
IRDye® 680RD Donkey anti-Mouse IgG (H + L)	LI-COR, Cat#926-68072	WB
IRDye® 680RD Donkey anti-Goat IgG (H + L)	LI-COR, Cat#926-68074	WB

IP, immunoprecipation; WB, western blot.

### Bacterial Strains and Cell Culture

*Streptococcus pneumoniae* (SPN) serotype 2 strain D39 (NCTC 7466), isogenic Δ*nanA* mutant, and *nanA*-complemented strains used in this study have been previously described (Chang et al., 2012). SPN was cultured in static liquid Todd-Hewitt broth (Acumedia) containing 2% yeast extract (Acumedia) at 37°C with 5% CO_2_ to mid-log phase for experiments. THP-1 cells (ATCC TIB-202), Siglec-5 overexpressing THP-1 cells (Sig-5/THP-1 cells, from Dr. Angata Takashi, Academia Sinica), and Siglec-5 knockdown THP-1 cells (Chang et al., 2012) were maintained in RPMI 1640 supplemented with 10% FBS, 10 mM HEPES, 1 mM sodium pyruvate, 2.5 g/L glucose, and 0.05 mM 2-mercaptoethanol. For some experiments, THP-1 cells were differentiated into macrophages with 25 ng/ml PMA for 24 h, followed by resting for 48 h in fresh RPMI 1640 medium before being used for experiments. Human primary monocytes were isolated from healthy donors (with use and procedures approved by the National Taiwan University IRB 201911067RINC) using MagniSort™ Human CD14 Positive Selection Kit (Thermo) and were differentiated to macrophages by culturing in RPMI 1640 medium supplemented with 10% FBS, 10 mM HEPES, 0.05 mM 2-mercaptoethanol, and 10 ng/ml M-CSF for 6 days. The *SIGLEC5/14* genotype of each donor was characterized by genomic PCR as previously described (Yamanaka et al., 2009), and macrophages derived from individuals with *SIGLEC14*-null genotype were used in this study.

### NanoString Gene Expression Analysis

THP-1 cells were infected with pneumococcus at a multiplicity of infection (MOI) of 10 for 6 h. RNA from infected cells was extracted by RNeasy Mini kit (Qiagen), followed by quantification for gene expression analysis using nCounter® human Immunology v2 panel (NanoString Technologies) through the service provided by Cold Spring Biotech, Taiwan. The generated gene expression data sets were analyzed by nSolver^TM^ analysis software (NanoString Technologies). Background values were corrected from raw data and normalized using 15 housekeeping genes (ABCF1, ALAS1, EEF1G, G6PD, GAPDH, GUSB, HPRT1, OAZ1, POLR1B, POLR2A, PPIA, RPL19, SHDA, TBP, and TUBB). Differential expressed genes (WT/Δ*nanA*>1 or <1) were further analyzed using the **d**atabase for **a**nnotation, **v**isualization and **i**ntegrated **d**iscovery (DAVID, https://david.ncifcrf.gov/).

### RNA Isolation, RT-PCR, and qRT-PCR

Total cellular RNA was extracted using NucleoZOL reagent (Macherey-Nagel) and transcribed to cDNA by PrimeScript RT reagent (TaKaRa) according to the manufacturer’s instructions. The resulted cDNA was amplified by quantitative RT-PCR using iQ^TM^ SYBR^®^ Green Supermix (Bio-rad) on the CFX96 Touch^TM^ real-time detection System (Bio-rad). Primers used for experiments were as follows: HPRT-1, CAAGCTTGCTGGTGAAAAGGAC, GTCAAGGGCATATCCTACAACAAA; IL-1β, AAATACCTGTGGCCTTGGGC, TTTGGGATCTACACTCTCCAGCT; IL-8, ATAAAGACATACTCCAAACCTTTCCAC, AAGCTTTACAATAATTTCTGTGTTGGC; TNF-α, CCCAGGGACCTCTCTCTAATCA, GCTTGAGGGTTTGCTACAACATG.

### Cytokine Detection

Released IL-1β, IL-8, and TNF-α in culture supernatants were quantified by enzyme-linked immunosorbent assay (ELISA) according to the manufacturer’s instructions (all from Invitrogen).

### Immunofluorescence Microscopy

THP-1 cells were seeded on poly-L-lysine coated coverslip in the presence of PMA at a final concentration of 25 ng/ml overnight. The differentiated THP-1 cells were infected with CFSE (carboxyfluorescein succinimidyl ester, BioLegend)-labeled pneumococcus at an MOI of 10 at 37°C for 1 h. Infected cells were fixed with fix solution (2% paraformaldehyde/PBS), permeabilized with 0.5% Triton X-100/fix solution, blocked with 3% BSA/PBS, and stained with biotin-conjugated ECA and Alexa Fluor 568-conjugated streptavidin (Thermo). Stained cells were counterstained with DAPI (4′,6-diamidino-2-phenylindole, Thermo) and visualized under fluorescent microscope (EVOS cell imaging system, Thermo).

### Isolation of Lipid Raft Fractions

THP-1 cells were infected with pneumococcus at an MOI of 10 for 1 h at 37 °C, rinsed with PBS, lysed with ice-cold 1% Brij-58/TNE buffer (25 mM Tris pH7.5, 150 mM NaCl and 5 mM EDTA) containing protease inhibitor cocktail (Roche), and kept on ice for at least 30 min. The raft-containing supernatants were collected by centrifuging at 300g at 4 °C for 5 min, gently mixed with an equal volume of 80% sucrose/TNE buffer, and centrifuged through a 5-30% continuous sucrose gradient in a SW41 Ti rotor (Beckman Coulter) at 40,000 rpm for 18 h at 4°C. Fourteen 0.7 ml fractions were sequentially collected from the top to bottom.

### Western Blotting and Co-Immunoprecipitation

THP-1 cells were infected with pneumococcus at an MOI of 10, 30, and 100 for 1 and 3 h. Infected THP-1 cells were lysed in 1% NP-40 lysis buffer containing Halt^TM^ protease and phosphatase inhibitor cocktail (Thermo) and centrifuged at maximal speed to collect cell lysates. Proteins released into the culture supernatants were precipitated with 25% trichloroacetic acid (TCA) at -80 °C overnight and pelleted by centrifugation. The resulted samples were resuspended with SDS-PAGE sampling buffer, separated on SDS-PAGE gels, transferred to PVDF membranes, detected with indicated primary antibodies and IRDye^®^ 800CW- or 680RD-conjugated secondary antibodies (Li-Cor), and visualized and quantified with a Li-Cor Odyssey scanner and software. For Co-immunoprecipitation, cells were lysed with 1% NP-40 lysis buffer with Halt^TM^ protease and phosphatase inhibitor cocktail. Cell lysates were incubated with indicated antibodies plus protein A/G mix magnetic beads (Millipore) at 4 °C overnight. The immunoprecipitates were washed extensively and resuspended with SDS-PAGE sampling buffer for western blot analysis. Densitometry of various analyte proteins and their respective loading controls from the same blot was performed using Image J 1.53 (NIH) software.

### Micro-Western Array (MWA)

THP-1 cells and human primary macrophages were infected with pneumococcus at an MOI of 5 at 37 °C for the indicated times. Lysates from infected cells were collected and subjected to Micro-Western Array analysis as previously described (Ciaccio et al., 2010). The resulted images were scanned by the Odyssey Infrared Imaging System (Li-Cor), quantified with Image Studio V5.2 software (Li-Cor), and normalized against β-actin.

### ASC Oligomerization Assay

THP-1 cells were infected with pneumococcus at an MOI of 30 and 100 for 1 and 3 h. The infected cells were resuspended in buffer A (20 mM Hepes-KOH, pH7.5, 10 mM KCl, 1.5 mM MgCl_2_, 1 mM EDTA, 320 mM sucrose) containing protease inhibitor cocktail, sheared by passing through 27G needles 10 times, and centrifuged at 1800 rpm for 8 min to remove intact cells and nuclei. The collected supernatant was mixed with an equal volume of CHAPS buffer (20 mM Hepes-KOH, pH 7.5, 5 mM MgCl_2_, 0.5 mM EGTA, 0.1% CHAPS) and centrifuged at 5000 rpm for 8 min. The resulted pellet was cross-linked with dextran sulfate sodium (Thermo, final concentration of 2 mM) for 30 min at room temperature and resuspended with SDS-PAGE sampling buffer for western blot analysis.

### WST-1 Assay

Cell viability was measured in triplicates by a colorimetric WST-1 kit (TaKaRa) according to manufacturer’s instructions. Briefly, THP-1 cells were cultured in phenol red-free RMPI medium containing 10% FBS and infected with pneumococcus at an MOI of 3, 10, and 30 for 3 h. The infected cells were incubated with WST-1 PreMix (10% of total volume) for 3 h at 37 °C. The absorbance of the samples was determined at 450 nm.

### Cell Death Analysis

THP-1 cells were infected with pneumococcus at an MOI of 30 for 1 h and 3 h, stained with APC-conjugated Annexin V and 7-ADD viability staining solution (BioLegend) for 15 min at room temperature in the dark, and immediately analyzed by FACS Calibur flow cytometer (BD).

### Apoptotic Nuclei Determination by Flow Cytometry

The percentage of apoptotic nuclei was measured by propidium iodine (PI) staining as previously described (Nicoletti et al., 1991). THP-1 cells were infected with pneumococcus at an MOI of 30 and 100 for 4.5 h, followed by fixation with 70% cold ethanol at -20 °C overnight. The fixed cells were stained with PI solution (PBS containing 20 μg/ml PI (Sigma-Aldrich), 400 μg/ml RNase A (Sigma-Aldrich), and 1% Triton X-100) for 30 min at room temperature in the dark. The PI fluorescence of individual nuclei was measured by FACS Calibur and analyzed with DNA analysis ModFit LT^TM^ software (Verity Software House).

### Statistics

All statistical tests were performed using GraphPad Prism version 8 software (GraphPad Software, Inc.). Differences were determined using the two-tailed t test, one-way ANOVA, or two-way ANOVA tests as indicated in the legend. A *p*-value <0.05 was considered statistically significant for all tests.

## Results

### Differentially Expressed Genes in Pneumococcus-Infected THP-1 Monocytes

**Figure 1:**
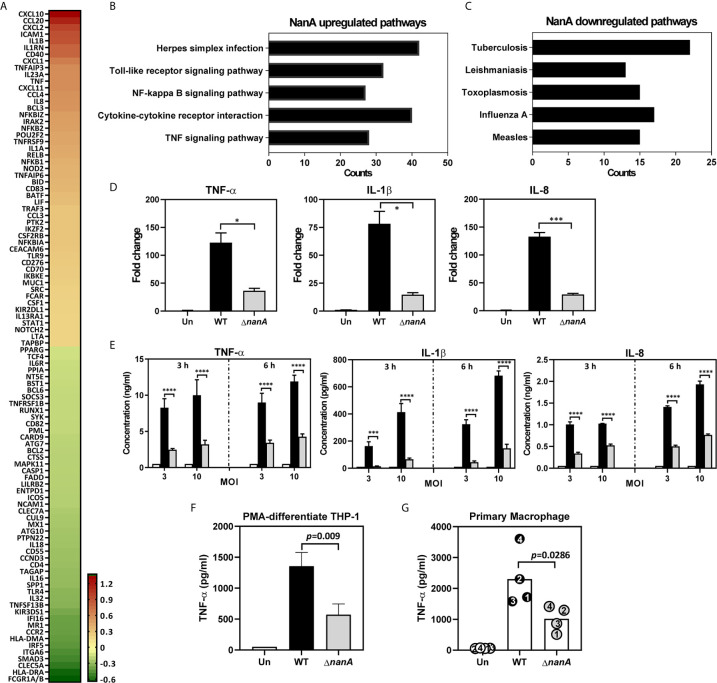
Major gene expression profile of THP-1 monocytes upon pneumococcal infection. **(A)** Heatmap of top 50 genes differentially expressed in WT SPN- and Δ*nanA*-infected Sig-5/THP-1 cells. Fold changes combined from two independent Nanostring experiments were calculated as the ratio of WT/ Δ*nanA*, followed by log2 transformation. A heatmap was created by Prism software. Top 5 pathways identified by KEGG pathway enrichment analysis of the **(B)** 220 NanA-upregulated genes and the **(C)**128 NanA-downregulated genes. **(D)** Parental THP-1 cells were infected with WT SPN and Δ*nanA* at an MOI of 10 for 3 h and the transcript levels of TNF-α, IL-1β, and IL-8 in the infected cells were analyzed by RT-qPCR analysis. Representative data from three independent experiments was shown as mean±SD. **(E)** Parental THP-1 cells were infected with WT SPN and Δ*nanA* at an MOI of 3 and 10 for 3 h and 6 h, and the released TNF-α, IL-1β, and IL-8 in the culture supernatant were measured by ELISA. The data presented as mean±SD was representative of three independent experiments, each performed in triplicate. **(F)** TNF-α concentration in supernatants was measured from PMA-differentiated THP-1 macrophages 3 h post pneumococcal infection (MOI of 10). Data represented mean±SD were pooled from two independent experiments, each performed in triplicate. **(G)** Primary human macrophages were infected with WT SPN and Δ*nanA* at an MOI of 10 for 3 h, and TNF-α concentration in supernatants was measured by ELISA. Each dot represented a different donor. Statistical analysis was performed using Student’s *t* test **(D–G)**. ****P < 0.0001; ***P < 0.001; *P < 0.05.

To achieve a comprehensive understanding of inflammation-related genes modulated by NanA and Siglec-5 in response to pneumococcal infection, we infected Siglec-5 overexpressing THP-1 cells (Sig-5/THP-1) with wild-type (WT) SPN or isogenic sialidase deficient mutant (Δ*nanA*) and profiled the expression of 579 immune‐related genes using the NanoString nCounter Human Immunology Panel. The complete list of the differentially expressed genes (DEGs, defined by WT/Δ*nanA* >1 or <1) between WT SPN- and Δ*nanA*-infected Sig-5/THP-1 cells was shown in **Table S1**. A total of 100 top differentially expressed genes, including 50 upregulated genes and 50 downregulated genes, were shown in **Figure 1A**. We found that NanA significantly upregulates the expression of proinflammatory and chemoattractant genes like IL1β, IL1RN, IL23A, IL8, TNF, CXCL1, CXCL2, CXCL10, CXCL11, CCL4, and CCL20 and genes involved in NF-κB signaling pathway, such as NFKB1, NFKB2, NFKB1A, NFKB1Z, and RELB. In contrast, surface receptors involved in pathogen recognition and antigen presentation, such as FCGR1A/B, HLA-DRA, CLEC5A, ITGA6, MR1, and CCR2, were downregulated by NanA. To identify the biological pathways targeted by pneumococcal NanA, we conducted Kyoto Encyclopedia of Genes and Genomes (KEGG) pathway analysis for all the DEGs identified from WT SPN- and Δ*nanA*-infected Sig-5/THP-1 cells. KEGG pathway analysis revealed that genes upregulated by NanA are highly enriched in pathways related to TLR, NF-κB, and cytokine/cytokine receptor (**Figure 1B** and **Table 2**). On the other hand, NanA-downregulated genes seemed to be involved in host responses related to viral infections, but these genes showed sporadic distribution and were not enriched in a given pathway (**Figure 1C** and **Table 3**).

**Table 2:** KEGG pathway analysis of genes upregulated by NanA.

KEGG ID	Description	Counts	P value (Benjamini)	Genes
hsa05168	Herpes simplex infection	42	7.61E-28	TRAF1, TRAF2, CCL2, TNF, TBK1, C3, C5, TLR2, TLR3, NFKBIA, NFKB1, CCL5, CD74, TLR9, CFP, CASP3, MYD88, TAP2, TICAM1, TAP1, IL1B, HLA-DOB, CHUK, LTA, IFNGR1, TRAF3, RELA, HLA-A, HLA-C, TNFRSF14, HLA-B, STAT1, IFNAR1, STAT2, IKBKE, IFNAR2, C1QBP, IKBKG, IL12A, JAK1, IRF3, IKBKB
hsa04620	Toll-like receptor signaling pathway	32	6.57E-25	CCL3, TNF, TBK1, TOLLIP, TLR1, TLR2, TLR3, NFKBIA, NFKB1, CXCL11, CCL5, CCL4, CXCL10, TLR9, IRAK4, MYD88, TICAM1, IL1B, CHUK, TRAF3, RELA, CD40, STAT1, IFNAR1, IKBKE, IFNAR2, CD86, CD80, IKBKG, IL12A, IRF3, IKBKB
hsa04064	NF-kappa B signaling pathway	27	2.92E-21	TRAF1, TRAF2, TNF, PTGS2, NFKBIA, NFKB1, NFKB2, CCL4, BTK, IRAK4, MYD88, TICAM1, IL1B, LTA, CHUK, TRAF3, ICAM1, BCL10, LTBR, RELA, RELB, TNFRSF13C, CD40, IKBKG, TNFAIP3, IKBKB, PLAU
hsa04060	Cytokine-cytokine receptor interaction	40	7.30E-21	CXCL1, CCL3, CCL2, TNF, IL6ST, CSF1, CXCL2, CXCR2, CD70, CXCR3, CXCL11, CCL5, CCL4, TGFB1, CXCL10, LIF, CCL22, IL23A, CCL20, CXCR4, CSF2RB, IL1B, IL13RA1, XCR1, LTA, IFNGR1, IL1A, IL18R1, LTBR, TGFBR2, TNFRSF13C, TNFRSF14, CD40, IL11RA, IFNAR1, IFNAR2, TNFRSF9, CCR7, CX3CR1, IL12A
hsa04668	TNF signaling pathway	28	6.57E-20	CXCL1, TRAF1, TRAF2, TNF, CCL2, PTGS2, CSF1, CXCL2, NFKBIA, NFKB1, CCL5, CXCL10, LIF, NOD2, CASP3, CCL20, BCL3, IL1B, LTA, CHUK, TRAF3, ICAM1, IL18R1, CEBPB, RELA, IKBKG, IKBKB, TNFAIP3

**Table 3:** KEGG pathway analysis of genes downregulated by NanA.

KEGG ID	Description	Counts	P value (Benjamini)	Genes
hsa05152	Tuberculosis	22	3.61E-14	IRAK1, CARD9, IL18, TLR4, FADD, MALT1, ITGB2, MAPK11, CTSS, HLA-DMA, MAPK1, IL10RA, BCL2, MAPK14, CASP8, FCER1G, JAK2, FCGR2A, CLEC7A, TRAF6, SYK, HLA-DRA
hsa05140	Leishmaniasis	13	2.54E-10	PTPN6, IRAK1, ITGB2, MAPK11, TLR4, ITGA4, HLA-DMA, MAPK1, MAPK14, JAK2, FCGR2A, TRAF6, HLA-DRA
hsa05145	Toxoplasmosis	15	3.79E-10	IRAK1, SOCS1, MAPK11, TLR4, HLA-DMA, TYK2, MAPK1, ITGA6, IL10RA, MAPK14, BCL2, CASP8, JAK2, TRAF6, HLA-DRA
hsa05164	Influenza A	17	2.56E-09	IFIH1, SOCS3, IL18, PML, TLR4, MAPK11, HLA-DMA, TYK2, MAPK1, TNFRSF10C, TNFSF10, IRF7, MAPK14, JAK2, MX1, CASP1, HLA-DRA
hsa05162	Measles	15	4.82E-09	TYK2, IRAK1, TNFRSF10C, IFIH1, TNFSF10, CCND3, STAT5A, IRF7, STAT5B, TP53, TLR4, JAK2, IL2RG, TRAF6, MX1

Overexpression of Siglec receptors in myeloid cells has been shown to play both a positive and a negative role in regulating the immune responses upon various stimulations (Ohta et al., 2010; Higuchi et al., 2016; Li et al., 2019). To ascertain that the profound changes observed in Sig-5/THP-1 cells are not attributed from Siglec receptor overexpression, we used quantitative reverse transcription-PCR (qRT-PCR) to examine the expression of NanA-upregulated inflammatory mediators in parental THP-1 cells which express only low levels of endogenous Siglec-5. In line with what has been shown in Sig-5/THP-1, NanA also remarkably increased the expression of inflammatory genes TNF-α, IL-1β, and IL-8 in pneumococcus-infected parental THP-1 cells (**Figure 1D**). In accordance with the gene expression data, a 3-fold increase in TNF-α, IL-1β, and IL-8 protein levels was found in the WT SPN-infected THP-1 cells compared to the Δ*nanA* -infected cells (**Figure 1E**). Similar findings were also observed for TNF-α production in the PMA-differentiated THP-1 macrophages (**Figure 1F**) and human primary macrophages (**Figure 1G**). Together, these results highlight a critical role of pneumococcal NanA in regulating the inflammatory responses in both THP-1 macrophages and human primary macrophages.

### NanA Dysregulates the Interaction of Siglec-5 With TLR2 and SHP-1

**Figure 2:**
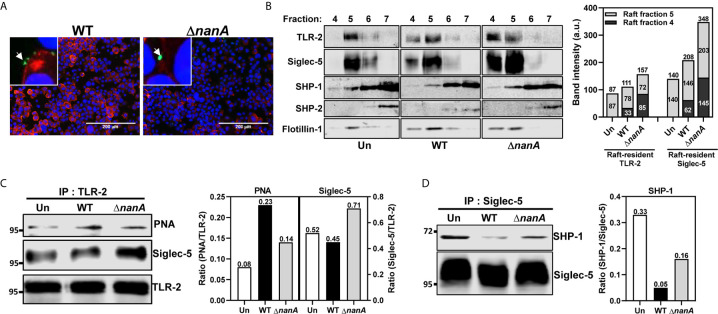
NanA dysregulated the interaction of Siglec-5 with TLR2 and SHP-1. **(A)** Surface desialylation analysis of WT SPN- and Δ*nanA*-infected THP-1 cells. THP-1 cells were infected with CFSE-labeled pneumococcus (green) at an MOI of 30 for 1 h followed by staining with biotin-conjugated ECA and Alexa Fluor 568-conjugated streptavidin (red). DNA was stained with DAPI (blue). **(B)** The distribution of TLR-2 and Siglec-5 in the lipid rafts. Sig-5/THP-1 cells were uninfected (Un) or infected with WT SPN and Δ*nanA* at MOI of 10 for 1 h. Cell lysates collected from infected cells were separated by sucrose-gradient ultracentrifugation and immunoblotting with Ab recognizing TLR2, Siglec-5, SHP-1, SHP-2, and flotillin-1. The summed band intensities of TLR-2 and Siglec-5 in the raft fractions 4 and 5 were quantified by Image J. **(C)** Sig-5/THP-1 cells were infected with WT SPN and Δ*nanA* at an MOI of 10 for 1 h. The sialylation level and Siglec-5-interaction of TLR-2 were analyzed by immunoprecipitating cell lysates with anti-TLR-2 Ab, followed by probing with PNA and anti-Siglec-5 Ab, respectively. Band intensities of PNA signal and co-precipitated Siglec-5 were quantified by Image J and normalized to TLR-2 values. **(D)** Sig-5/THP-1 cells were infected with WT SPN and Δ*nanA* at an MOI of 10 for 40 min and the SHP-1 recruitment to Siglec-5 was examined by immunoprecipitating cell lysates with anti-Siglec-5 Ab, followed by probing with anti-SHP-1 Ab. Band intensities of co-precipitated SHP-1 were measured by Image J and normalized Siglec-5 values. The data shown in **Figure 2** were representative of two independent experiments.

TLR-2 is the major surface PRR responsible for recognizing a wide range of Gram-positive bacterial cell wall constituents, including peptidoglycans, lipopeptides, and lipoteichoic acids, to initiate host antibacterial responses (Yoshimura et al., 1999; Koedel et al., 2003; Schröder et al., 2003; Basset et al., 2013). TLR agonist stimulation has been shown to trigger lateral mobilization of TLR and Siglec receptors into a specialized cholesterol-enriched lipid raft domain, a crucial platform organizing surface receptors and related intracellular signaling molecules (Triantafilou et al., 2002; Munro, 2003; Triantafilou et al., 2006; Sezgin et al., 2017). It has been recently reported that the lectin activity (i.e. the sialic acid-binding activity) of Siglec is required for its raft translocation in response to TLR ligand stimulation (Ando et al., 2015). To examine whether the surface sialylation levels was reduced in the bacterial contact sites, we infected THP-1 cells with WT SPN and Δ*nanA* mutant and stained the infected cells with *Erythrina cristagalli* lectin (ECA) which preferentially recognizes uncapped glycans without terminal sialic acids. We found that the ECA signals clearly increase on the THP-1 surface where WT SPN contacts. In contrast, Δ*nanA*-infected cells showed weak or no ECA signals (**Figure 2A**). Given that NanA reduces the surface sialylation of infected cells and binding of Siglec to its sialyl ligands is necessary for its raft localization, we then investigated whether NanA has any effect on the translocation of TLR-2 and Siglec-5 into lipid rafts upon pneumococcal infection. Consistent with the previous report that a substantial amount of TLR-2 is raft-resident without agonist stimulation (Snodgrass et al., 2013), TLR-2 was detected in the raft fraction 5, indicated by the raft marker flotillin-1, isolated from unstimulated THP-1 cells (**Figure 2B**). Upon pneumococcal infection, a considerate amount of Siglec-5 and TLR-2 were recruited to the raft fractions 4 and 5, and a significantly reduced raft translocation of Siglec-5 was observed in the WT SPN-infected cells (**Figure 2B**).

To determine whether the interaction between Siglec-5 and TLR-2 was sensitive to NanA-mediated desialylation, we infected Sig-5/THP-1 cells with WT SPN or Δ*nanA* mutant and examined the sialylation level of immunoprecipitated TLR-2 with peanut agglutinin (PNA) which detects the galactosyl-β1,3-*N*-acetylgalactosamine structure that normally appears after removal of the terminal sialic acids. PNA blotting demonstrated that TLR-2 precipitated from WT SPN-infected cells was less sialylated than TLR-2 from Δ*nanA*-infected cells (**Figure 2C**). In addition, co-precipitation of Siglec-5 by anti-TLR-2 antibodies was remarkably reduced in WT SPN-infected cells compared to Δ*nanA*-infected cells (**Figure 2C**). Siglecs are known to negatively regulate immune responses via recruiting SHP phosphatases to suppress tyrosine kinase-dependent signals (Crocker et al., 2007; Pillai et al., 2012). Reduced recruitment of SHP-1 to Siglec-5 was observed in WT SPN-infected but not Δ*nanA*-infected THP-1 cells (**Figure 2D**). These results indicate that the sialyl-sugar residues of TLR-2 are important for its interaction with Siglec-5 and this sialic acid-dependent interaction is sensitive to NanA-mediated desialylation.

### NanA Enhances the Activation of Multiple Inflammation-Related Signaling Molecules

**Figure 3:**
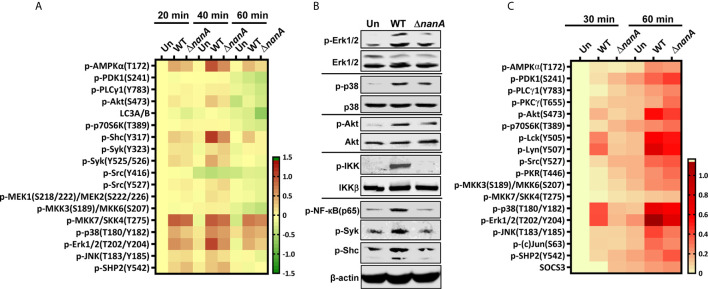
Inflammation-related signaling molecules modulated by NanA upon pneumococcal infection. **(A)** THP-1 cells were infected with WT SPN and Δ*nanA* at an MOI of 5 for 20, 40, and 60 min. The cell lysates were collected and subjected to Micro-Western analysis (MWA). Image density was quantified by Image Studio software and normalized to β-actin. Fold change was calculated as the ratio of each normalized net intensity to the net intensity of the uninfected control, followed by log2 transformation. The heatmap was created by Prism software. **(B)** Western blot verification of the phosphorylation of signaling molecules revealed by MWA. Cytoplasmic proteins were extracted from WT SPN- and Δ*nanA*-infected THP-1 cells and subjected to western blotting for detection of the phosphorylated Erk1/2, p38, Akt, IKK, NF-κB p65, Syk, and Shc. **(C)** Human primary macrophages were infected with WT SPN and Δ*nanA* at an MOI of 5 for 30 and 60 min, and the activation of inflammation-related signaling molecules was examined in by MWA as described in **(A)**.

Pneumococcus was known to activate multiple TLRs and NLRs to induce a range of inflammatory responses (Koedel et al., 2003; Malley et al., 2003; Knapp et al., 2004; Opitz et al., 2004; Mcneela et al., 2010; Davis et al., 2011; Witzenrath et al., 2011). To further investigate the mechanistic role of NanA in PRR signaling pathways, we used micro-western array (MWA) and regular western blot analysis to examine the phosphorylation level of numerous downstream signaling proteins of PRRs. As shown in **Figures 3A, B**, the phosphorylation level of signaling proteins belonging to the NF-κB and MAPK pathways, such as IKK, NF-κB(p65), MKK7/SKK4, p38 MAPK, Erk1/2, and JNK, was markedly more upregulated in WT SPN-infected cells than in Δ*nanA*-infected cells (**Figures 3A, B**).

In addition, several proteins acting more upstream in the PRR signaling cascade, such as AMPKα, Akt, Shc, and Syk, showed higher phosphorylation levels in WT SPN-infected cells (**Figures 3A, B**). AMPKα has been shown to regulate multiple inflammatory pathways, including NK-κB, JNK, and NLRP3 inflammasome (Moon et al., 2015; Gaber et al., 2017; Silwal et al., 2018). Akt plays a critical role in the induction of the transcriptional activity of NF-κB (Kane et al., 1999; Ozes et al., 1999). Shc is a key adaptor protein known to activate the MAPK pathway in response to various stimulation (Ravichandran, 2001). Syk has been shown to phosphorylate MyD88 and ASC to regulate IL-1β-driven inflammation and NLRP3 inflammasome-mediated caspase-1 activation, respectively (Lin et al., 2015; Gurung et al., 2017; Feng et al., 2018). These observations suggest that NanA may target the upstream regulators of the PRR signaling pathway to exaggerate inflammation upon pneumococcal infection.

On the other hand, phosphorylation of Src at tyrosine 527 (Y527) negatively regulates its kinase activity by locking Src in a closed conformation (Roskoski, 2005; Byeon et al., 2012). Phosphorylation of SHP-2 at tyrosine 542 (Y542) is required for its phosphatase activity, which negatively regulates TLR-induced immune responses (An et al., 2006). Elevated phosphorylation of Src and SHP-2 at Y527 and Y542, respectively, were more pronounced in Δ*nanA*-infected cells (**Figure 3A**), which suggests that negative regulators in the PRR signaling pathway may be repressed in the presence of NanA upon pneumococcal infection. Notably, a similar phosphorylation profile of the TLR-related kinases and phosphatases was observed in WT SPN- and Δ*nanA*-infected primary human macrophages, which more closely resembles the phenotype of healthy cells *in vivo* (**Figure 3C**). Taken together, our data suggest that kinases and phosphatases may be differentially regulated by NanA to broadly exaggerate inflammatory signals emanating from PRRs.

### NanA-Mediated Caspase-1 and NLRP3 Inflammasome Activation Causes the Excessive IL-1β Production

**Figure 4:**
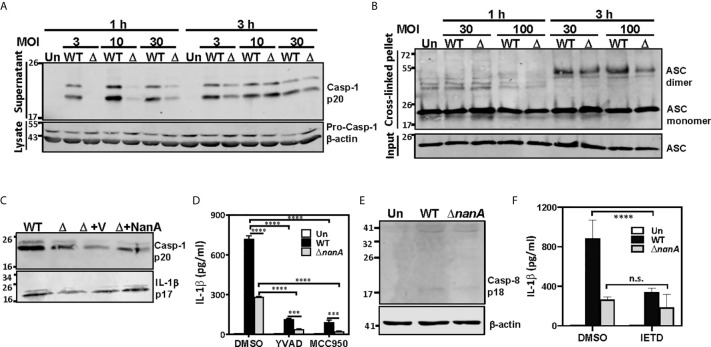
NanA-mediated caspase-1 and NLRP3 inflammasome activation causes excessive IL-1β production. **(A)** THP-1 cells were infected with WT SPN and Δ*nanA* at an MOI of 3, 10 and 30 for 1 and 3 h, and the supernatant and cell lysates were analyzed by western blotting with anti-caspase-1 Ab. **(B)** NanA promotes ASC oligomerization. THP-1 cells were infected with WT SPN or Δ*nanA* mutants at an MOI of 30 and 100 for 1 h and 3 h. The cell lysates and DSS cross-linked pellets prepared from infected cells were subjected to western blotting to analyze ASC oligomerization. **(C)** THP-1 cells were infected with WT SPN, Δ*nanA*, and NanA-complemented strains at an MOI of 10, and the supernatant was collected 1 h and 8 h post-infection and analyzed by western blotting with anti-caspase-1 and anti-IL-1β Ab, respectively. **(D)** THP-1 cells were treated with caspase-1 (Ac-YVAD-cmk) and NLRP3 (MCC950) inhibitors at 20 μM and 10 μM, respectively, for 1 h before infection with WT SPN or Δ*nanA* mutants at an MOI of 10 for 3 h. Culture supernatants were collected and quantified for IL-1β by ELISA. The data presented as mean±SD is representative of three independent experiments performed with biological triplicates. **(E)** THP-1 cells were infected with WT SPN and Δ*nanA* at an MOI of 30 for 8 h, and the cell lysate was analyzed by immunoblotting with anti-caspase-8 Ab. **(F)** THP-1 cells were treated with caspase-8 inhibitor (Z-IETD-FMK) at 20 μM for 1 h before infection with WT SPN or Δ*nanA* mutants at an MOI of 10 for 3 h. Culture supernatants were collected and quantified for IL-1β by ELISA. The data presented as mean±SD is representative of three independent experiments performed with biological triplicates. Statistical analysis was performed using two-way ANOVA **(D, E)**. ****P < 0.0001; ***P < 0.001; n.s., not significant.

In addition to the proinflammatory cytokines which secrete immediately following their transcription and translation, we noted that NanA strongly promotes the production of IL-1β which requires additional proteolytic maturation steps (**Figure 1**). The canonical cleavage and process of pro*-*IL*-*1β to mature IL-1β is catalyzed by caspase-1 (Franchi et al., 2009); thus, we tested whether NanA promotes caspase-1 activation upon pneumococcal infection. THP-1 cells were infected with WT SPN and Δ*nanA*, and the culture supernatant was collected from infected cells, TCA-precipitated, and probed with anti-caspase-1 antibodies. In accordance with the excessive IL-1β production in WT SPN-infected THP-1 cells, elevated caspase-1 activation, as indicated by the increased amount of cleaved p20 subunits of caspase-1, was observed in WT SPN-infected THP-1 cells compared to Δ*nanA*-infected cells (**Figure 4A**). Given that assembly of the multimeric protein complex known as inflammasome, which comprises members of the NLR family, apoptosis-associated speck-like protein containing a caspase recruitment domain (ASC), and pro-caspase-1, is a prerequisite step for caspase-1 cleavage (Franchi et al., 2009), we then examined the role of NanA in ASC oligomerization, a hallmark of inflammasome activation. As shown in **Figure 4B**, greater levels of ASC oligomerization were induced in WT SPN-infected THP-1 cells than in Δ*nanA*-infected cells. These findings indicate that NanA positively regulates the formation of inflammasome complexes and subsequent caspase-1 activation.

To verify the role of NanA on caspase-1 and IL-1β maturation, we analyzed the levels of cleaved IL-1β p17 fragments in culture supernatants collected from infected THP-1 cells by western blot. As shown in **Figure 4C**, higher levels of cleaved caspase-1 p20 and IL-1β p17 were detected in THP-1 cells infected with SPN WT, although complementation of the Δ*nanA* mutant with NanA expressed on a plasmid only moderately increased the production of active caspase-1 and IL-1β. This partial complementation phenotype may be attributed to different NanA levels expressed from the plasmid in *trans* or driven by its native promoter. To understand whether the production of IL-1β is dependent on the activation of canonical NLRP3-ASC-caspase-1 inflammasome, we tested the effect of specific inhibitors targeting caspase-1 (Ac-YVAD-cmk) and NLRP-3 inflammasome (MCC950). Enhanced IL-1β release seen in WT SPN-infected cells was drastically reduced in the presence of caspase-1 and NLRP3 inflammasome inhibitors (**Figure 4D**), which supports the critical role of NLRP3 inflammasomes in NanA-augmented IL-1β secretion. However, small amounts of IL-1β were still detectable in the WT SPN-infected THP-1 cells in the presence of NLRP3 inflammasome or caspase-1 inhibitors, indicating that an alternative mechanism for IL-1β maturation may exist. Given that caspase-8 has been identified as an alternative protease to mediate atypical pro-IL-1β processing (Maelfait et al., 2008; Gringhuis et al., 2012), we thought to verify the role of caspase-8 in NanA-augmented IL-1β production. A small amount of cleaved caspase-8 p18 fragments was detected in WT SPN-infected THP-1 cells (**Figure 4E**), and caspase-8 inhibitors (Z-IETD-FMK) moderately reduced the excessive IL-1β secretion seen in WT SPN-infected THP-1 cells (**Figure 4F**). These data indicate that the canonical NLRP3-caspase-1 inflammasome is responsible for most of the NanA-mediated excessive IL-1β production upon pneumococcal infection. The noncanonical caspase-8 may also contribute to the NanA-mediated excessive IL-1β production, although further experiments will be required to delineate the role of caspase-8 in IL-1β production upon pneumococcal infection.

### Multiple Death Pathways Are Involved in NanA-Exacerbated Cell Death Upon Pneumococcal Infection

**Figure 5:**
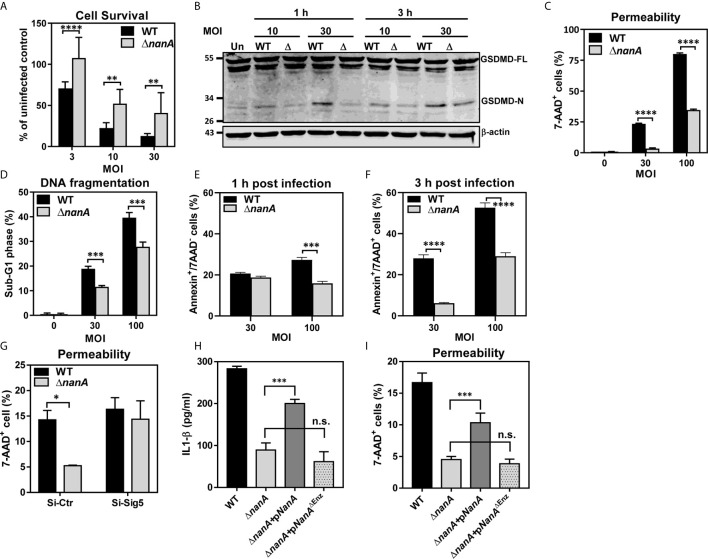
Multiple death pathways contributed to NanA-exacerbated cell death upon infection **(A)** THP-1 cells were infected with WT SPN and Δ*nanA* at an MOI of 3, 10, and 30 for 3 h. The infected cells were further cultured with premix WST-1 reagent for 3 h. The absorbance was determined at 450 nm and the background values were subtracted. Results were presented as percentages of the uninfected control. Data represented mean±SD pooled from three independent experiments, each performed in triplicate. **(B)** THP-1 cells were infected with WT SPN and Δ*nanA* at an MOI of 10 and 30 for 1 and 3 h, and cell lysates were harvested for western blotting with antibodies for GSDMD and β-actin. **(C)** Flow cytometric analysis of 7-AAD uptake. THP-1 cells were infected with WT SPN and Δ*nanA* at an MOI of 30 and 100 for 3 h, and the infected cells were stained with 7-AAD to analyze the membrane integrity of the infected cells. **(D)** THP-1 cells were infected with WT SPN and Δ*nanA* at an MOI of 30 and 100 for 4.5 h. The DNA content of infected cells was revealed by flow cytometric analysis of propidium iodide-stained nuclei. Flow cytometric analysis of cell apoptosis. THP-1 cells were infected with WT SPN and Δ*nanA* at an MOI of 30 for 1h **(E)** and 3 h **(F)**, and the infected cells were stained with Annexin V apoptosis detection kit with 7-AAD. **(G)** Siglec-5 knockdown (Si-Sig5) and control knockdown (Si-Ctr) THP-1 cells were infected with WT SPN and Δ*nanA* at MOI of 30 for 3 h, and the infected cells were stained with 7-AAD to analyze the membrane integrity of the infected cells. IL-1β concentration in supernatants **(H)** and cell pyroptosis **(I)** were analyzed 3 h post-infection in THP-1 cells challenged with WT SPN, Δ*nanA*, or the Δ*nanA* complemented with NanA or enzymatically inactive NanA (NanA^ΔEnz^) expression plasmids. The data shown were representative of two independent experiments **(B–I)**. Statistical analysis was performed using Student’s *t* test **(A, C–I)**. ****P < 0.0001; ***P < 0.001; **P < 0.01; n.s., not significant.

Bacterial infection often elicits substantial inflammation and cell death in the host. To examine whether NanA also promotes the cell death of pneumococcus-infected cells, we first used a WST-1-based cell cytotoxicity assay to measure the overall viability of THP-1 cells infected with WT SPN or Δ*nanA* mutants. As shown in **Figure 5A**, pneumococcus induced pronounced cell death in THP-1 cells within 3 h in a dose-dependent manner, and more reduced viability was observed in WT SPN-infected cells than in Δ*nanA*-infected cells at all tested MOI. Both pyroptosis and apoptosis have been reported to contribute to pneumococcus-infected cell death (Aliprantis et al., 1999; Srivastava et al., 2005; Bewley et al., 2014; Kim et al., 2015). Pyroptosis is a form of programmed cell death driven by the activation of inflammatory caspases. Characteristic features of pyroptosis include rapid plasma-membrane rupture, release of cytosolic contents, and DNA fragmentation (Bergsbaken et al., 2009; Miao et al., 2011). Caspase-1 has been shown to trigger pyroptosis by proteolytic cleavage of gasdermin D (GSDMD) to generate a N-terminal fragment (GSDMD-N) that forms membrane pores and ultimately causes cell lysis (Shi et al., 2015). Since we have shown that NanA increases caspase-1 activation, the generation of GSDMD-N was further examined in pneumococcus-infected cells. In accordance with the increased caspase-1 activity in WT SPN-infected THP-1 cells shown in **Figure 4A**, higher levels of GSDMD-N were detected in the WT SPN-infected cells than in Δ*nanA*-infected cells at all tested MOIs (**Figure 5B**). The membrane permeability which was determined by the penetration of membrane impermeable dye 7-AAD into cells was also drastically increased in the WT SPN-infected THP-1 cells (**Figure 5C**). In addition, THP-1 cells challenged with WT SPN resulted in higher numbers of cells in the sub-G1 phase which is indicative of DNA fragmentation, a key feature of apoptosis and pyroptosis (**Figure 5D**). To further examine whether NanA enhances the apoptosis of pneumococcus-infected THP-1 cells, we stained the infected cells with Annexin V/7-AAD apoptosis detection reagents. As shown in **Figures 5E, F**, there were more early (Annexin V^+^/7-AAD^-^) and late apoptotic cells (Annexin V^+^/7-AAD^+^) detected in WT SPN-infected cells than in Δ*nanA*-infected cells. Our data suggest that NanA-exacerbated cell death was mainly caused by the inflammatory caspase-mediated pyroptosis, while a minority of cell death was possibly mediated by apoptosis.

To further determine the relevance of Siglec-5 in NanA-potentiated pyroptosis, the membrane integrity was determined in the control (Si-Ctr) and Siglec-5 knockdown (Si-Sig5) THP-1 cells following SPN WT and Δ*nanA* mutants challenge. As shown in **Figure 5G**, pronounced cell pyroptosis was observed in WT-infected Si-Ctr THP-1 cells compared to Δ*nanA*-infected cells, while both WT and Δ*nanA* mutant induced pronounced pyroptosis in Si-Sig5 THP-1 cells. In addition, complementation of the Δ*nanA* mutant with the NanA enzyme expressed on a plasmid vector partially restored the released IL-1β (**Figure 5H**) and cell pyroptosis (**Figure 5I**). In contrast, complementation of the Δ*nanA* mutant with an enzymatically inactive version of NanA (NanA^ΔEnz^) had no effect. Our findings suggested that NanA-mediated surface desialylation possibly abrogates the Siglec-5-TLR-2 interaction to release the Siglec-5-mediated inhibitory signals, which was attributed to the elevated IL-1β release and cell pyroptosis in pneumococcus-infected cells.

## Discussion

Upon infection, immune cells sense the environment through their PRRs and integrate this external information through intracellular signaling molecules to launch robust inflammatory and antimicrobial responses to defend against microbial infection (Janeway and Medzhitov, 2002). In general, a fine-balanced immune response which is sufficient to eliminate pathogens but not too overactive so as to cause widespread host tissue damage is achieved through an intricate interaction between activating and inhibitory receptors (Lee and Kim, 2007; Zak and Aderem, 2009). In this study, we demonstrated that NanA, a virulence factor expressed by all pneumococcal isolates, caused extensive surface desialylation of the infected cells, which in turn impairs the sialic acid-dependent interaction between Siglec-5-TLR-2 and subsequent SHP-1 phosphatase recruitment. Thus, this NanA-dysregulated crosstalk between TLR-2 and inhibitory Siglec-5 exaggerated multiple inflammatory and death signaling pathways and caused excessive inflammation and cytotoxicity in pneumococcus-infected macrophages.

Recognition of bacterial components by the innate immune system is essential for the host to defend against invading pathogens. Many surface and cytosolic PRRs have been shown to recognize numerous pneumococcal components, such as peptidoglycans, teichoic acids, genomic DNAs, and pneumolysins, to initiate a protective innate immune response. Although this mechanism ensures the activation of immune cells upon pneumococcal infection, it may risk affecting the host with overwhelming inflammation when the cell activation goes uncontrolled. Siglecs are membrane-bound lectins that recognize the sialic acid-containing structures. Most of the Siglecs contain cytosolic ITIM- or ITIM-like motifs and are considered to play a negative role in cell activation via associating with tyrosine phosphatases to dephosphorylate key kinases or signaling proteins responsible for cell activation (Crocker et al., 2007; Pillai et al., 2012). A broad and direct interaction between TLR and Siglec was identified where Siglec negatively regulates TLR activation in response to TLR agonist stimulation (Chen et al., 2014). In addition, binding of CD14, a co-receptor for TLR-4, to Siglec-3 also downregulated the LPS-mediated TLR-4 activation (Ishida et al., 2014). These observations indicate that the Siglec-interacting property of TLRs may add an extra assurance to prevent over-activation of TLRs in response to ligand stimulation.

Dimerization and translocation of TLR-2 to lipid rafts, a specialized membrane microdomain organizing surface receptors and intracellular signaling molecules, was required for its activation and signal transduction (Triantafilou et al., 2006; Ruysschaert and Lonez, 2015). Although a comparable amount of TLR-2 was recruited to the lipid rafts upon WT SPN and Δ*nanA* stimulation, reduced translocation of Siglec-5 to the lipid rafts was clearly observed in the WT SPN-infected cells. This discoordinated surface distribution of TLR-2 and Siglec-5 was further evidenced by the reduced co-immunoprecipitation of Siglec-5 by anti-TLR-2 antibodies (**Figure 2**). This decoupled Siglec-5/TLR-2 interaction contributes, at least in part, to the exaggerated activation of multiple inflammation-related signaling molecules and subsequent excessive inflammation in pneumococcus-infected THP-1 cells (**Figures 1** **–** **3**).

The primary role of the inflammatory cytokines and chemotactic chemokines released upon microbial infection is to drive the maturation, homing, and activation of immune cells, which often attributes to enhanced microbicidal activities (Commins et al., 2010). Paradoxically, all identified pneumococcal isolates express NanA, which has been shown to exaggerate inflammatory responses upon infection (Chen et al., 2011; Chang et al., 2012). There are several possible explanations for this discrepancy. First, NanA may have an indispensable function in pneumococcal physiology and pathogenesis, which is evident by its critical role in nutrient acquisition, biofilm formation, and host colonization (Tong et al., 2002; Manco et al., 2006; Parker et al., 2009; Uchiyama et al., 2009). Second, inflammation normally occurs to alarm and boost the host immune responses to eradicate invading pathogens. However, the same response may cause undesired collateral tissue damage, which in turn facilitates bacterial dissemination (Chen et al., 2011; Chang et al., 2012). Here, we identified a new virulent role of NanA in exaggerating the cell death of immune cells as a means to subvert host antimicrobial responses (**Figure 5**). Several mechanisms have been suggested for pneumococcus-induced cell death, mostly through the activation of inflammasomes and caspase cascades (Aliprantis et al., 1999; Srivastava et al., 2005; Bewley et al., 2014; Gonzalez-Juarbe et al., 2015; Kim et al., 2015). In this study, we found that NanA-mediated desialylation enhances ASC oligomerization, caspase-1 activation, and GSDMD proteolytic cleavage in infected THP-1 cells (**Figures 4A, B**, and **5B**). In line with these observations, enhanced pyroptosis was observed in the infected THP-1 cells in the presence of NanA (**Figure 5**). Siglec-mediated modulation of inflammasome activation has also been reported in NK cells where human neonatal pathogen group B *Streptococcus* (GBS) suppresses NLRP3 inflammasome activation and prevents subsequent pyroptotic cell death through engaging ITIM-containing Siglec-7 in NK cells (Fong et al., 2018). In contrast, GBS triggered NLRP3 inflammasome activation in THP-1 cells overexpressing ITAM-coupling Siglec-14 whereas the same bacteria inhibited NLRP3 inflammasome activation when THP-1 cells expressed ITIM-containing Siglec-5 (Tsai et al., 2020). These observations suggest that bacterial species may manipulate inflammasome activation through engaging Siglecs (Fong et al., 2018; Tsai et al., 2020) or releasing Siglec-restricted inhibition (our studies), although further studies are required for a better understanding of how Siglec acts on the NLRP3-caspase-IL-1β axis.

In conclusion, our results reveal a novel virulence role of NanA in pneumococcal pathogenesis and suggest that targeting NanA activity may ameliorate the pneumococcus-mediated inflammation and cytotoxicity in severe invasive pneumococcal diseases.

## Data Availability Statement

The original contributions presented in the study are publicly available. This data can be found here: GEO repository with accession number GSE161269 (https://www.ncbi.nlm.nih.gov/geo/query/acc.cgi?acc=GSE161269).

## Ethics Statement

The studies involving human participants were reviewed and approved by National Taiwan University IRB 201911067RINC. The patients/participants provided their written informed consent to participate in this study.

## Author Contributions

Y-WT, C-CC, and Y-CC conceived and designed the experiments. Y-WT and C-CC performed the experiments and analyzed the data. Y-WT and Y-CC wrote the paper. All authors contributed to the article and approved the submitted version.

## Funding

This work was supported by grants from Taiwan MOST 106-2320-B-002-013-MY3 and 109-2320-B-002-058 and National Taiwan University NTU-CC-110L890502 to Y-CC.

## Conflict of Interest

The authors declare that the research was conducted in the absence of any commercial or financial relationships that could be construed as a potential conflict of interest.

## Supplementary Material

Supplementary Table 1 Differentially expressed genes between WT SPN- and Δ*nan*A mutant-infected Sig-5/THP-1 cells.
